# A Novel Role of Prolidase in Cocaine-Mediated Breach in the Barrier of Brain Microvascular Endothelial Cells

**DOI:** 10.1038/s41598-018-37495-6

**Published:** 2019-02-22

**Authors:** Binah baht Ysrayl, Muthukumar Balasubramaniam, Ife Albert, Fernando Villalta, Jui Pandhare, Chandravanu Dash

**Affiliations:** 1 Center for AIDS Health Disparities Research, Meharry Medical College, Nashville, Tennessee USA; 2 Center for Molecular and Behavioral Neurosciences, Meharry Medical College, Nashville, Tennessee USA; 3 School of Graduate Studies and Research, Meharry Medical College, Nashville, Tennessee USA; 4 Department of Microbiology, Immunology, and Physiology, Meharry Medical College, Nashville, Tennessee USA; 5 Department of Biochemistry, Cancer Biology, Neuroscience and Pharmacology, Meharry Medical College, Nashville, Tennessee USA

## Abstract

Cocaine use is associated with breach in the blood brain barrier (BBB) and increased HIV-1 neuro-invasion. We show that the cellular enzyme “Prolidase” plays a key role in cocaine-induced disruption of the BBB. We established a barrier model to mimic the BBB by culturing human brain microvascular endothelial cells (HBMECs) in transwell inserts. In this model, cocaine treatment enhanced permeability of FITC-dextran suggesting a breach in the barrier. Interestingly, cocaine treatment increased the activity of matrix metallo-proteinases that initiate degradation of the BBB-associated collagen. Cocaine exposure also induced prolidase expression and activity in HBMECs. Prolidase catalyzes the final and rate-limiting step of collagen degradation during BBB remodeling. Knock-down of prolidase abrogated cocaine-mediated increased permeability suggesting a direct role of prolidase in BBB breach. To decipher the mechanism by which cocaine regulates prolidase, we probed the inducible nitric oxide synthase (iNOS) mediated phosphorylation of prolidase since mRNA levels of the protein were not altered upon cocaine treatment. We observed increased iNOS expression concurrent with increased prolidase phosphorylation in cocaine treated cells. Subsequently, inhibition of iNOS decreased prolidase phosphorylation and reduced cocaine-mediated permeability. Finally, cocaine treatment increased transmigration of monocytic cells through the HBMEC barrier. Knock-down of prolidase reduced cocaine-mediated monocyte transmigration, establishing a key role of prolidase in cocaine-induced breach in endothelial cell barrier.

## Introduction

The Central Nervous System (CNS) is a major target of HIV-1^1^. The virus enters the brain during the early phase of infection and causes neuronal damage^2–4^ and a battery of deficits termed as HIV-associated neurological disorder (HAND)^5–7^. Entry of HIV-1 into the brain is facilitated by a “Trojan Horse” mechanism, where infected CD4+ cells and/or monocytes are trafficked into the CNS by penetrating through the blood brain barrier (BBB)^8,9^. Cocaine, a commonly used drug among HIV patients^10^, has been associated with worsening of HAND^11–16^. Although the exact mechanism remains unclear, it has been suggested that cocaine exposure enhances HIV-1 neuro-invasion by breaching the BBB^17–19^.

The main function of the BBB is to protect the brain by regulating the transport of substances between the peripheral circulation and the CNS^20^. The protective structure of BBB is formed primarily by the specialized endothelial cells along with pericytes, and astrocytic foot processes^20–23^. Additionally, the impenetrability of endothelial cells is imparted by a continuous network of trans-membranous tight junction proteins that are connected to the actin cytoskeleton via intracellular zonula occludens-1 (ZO-1) proteins^20–23^. Interestingly, cocaine has been reported to alter expression of tight junction and other proteins associated with the endothelial barrier. For example, cocaine exposure resulted in the loss or modulation of tight junction proteins such as ZO-1^24^. Additionally, cocaine’s ability to alter the expression of intracellular adhesion molecule 1 (ICAM-1), vascular cell adhesion molecule 1 (VCAM-1), and endothelial-leukocyte adhesion molecule (ELAM or selectin-1) has been postulated as a key contributory factor for the ensuing BBB breach^17–19^. Accordingly, these biochemical alterations have been associated with increased leukocyte migration across the BBB, elevated levels of pro-inflammatory cytokines and chemokines such as TNF-α, nuclear factor kappa B (NF-kB), IL-6, and others, ultimately resulting in neuro-inflammation^18,25^. Cocaine also binds to its cognate receptor σ-1-R in HBMECs to induce expression of platelet-derived growth factor (PDGF) that plays important role in endothelial permeability^26^. Furthermore, cocaine upregulates the pro-migratory CCL2/CCR2 system, enabling the HIV-infected cells to cross the BBB^24^. Collectively, these studies suggest that alterations in tight junction accompanied by elevated levels of pro-inflammatory response by cocaine can compromise the integrity of the BBB and enhance HIV-1 neuro-invasion^27^.

Surprisingly, very little is known about the effects of cocaine on the extracellular matrix (ECM) component of the BBB^28,29^. ECM plays key roles in maintaining BBB integrity by surrounding and supporting the cellular components of the barrier^28,29^. Endothelial cells and astrocytes secrete the ECM proteins (collagens, proteoglycans, and glycoproteins) to generate and maintain the basement membranes (BMs) of the BBB^28,30^. ECM remodeling and reorganization is regulated by a family of matrix metalloproteinases (MMPs)^31–33^. Because remodeling of the ECM is central to BBB function^28,29^, MMPs play key roles in neurodegenerative diseases^34,35^. For example, MMP-7 and MMP-9 are involved in the breakdown of the BBB in multiple sclerosis^36^. Both animal models and human studies have established a role of MMP-9 in BBB disruption in neuroinflammatory diseases^37–40^. Moreover, increased serum MMP levels have been reported in stroke patients^41–43^ and increased brain MMP activity during reperfusion^44,45^. Interestingly, reorganization of the ECM by MMP-2 and MMP-9 has been reported in cocaine addiction and relapse^46,47^. Cocaine treatment has also been shown to increase transcription of membrane type (MT)-MMP-1 in HBMECs^48^.

Interestingly, the degradation of ECM by the enzymatic activity of MMPs results in the accumulation of imidodipeptides and imidotripeptides with C-terminal proline or hydroxyproline^49,50^. These proline-containing short peptides are substrates of a special type of MMP-Prolidase^49,50^. Prolidase is a manganese-dependent cytosolic exopeptidase that plays an important role in ECM remodeling^49,50^. Given that ECM is critical for BBB function and prolidase regulates ECM degradation, we tested the role of prolidase in cocaine-induced dysfunction at the BBB. First we established an endothelial barrier model using HBMEC cells cultured on transwell supports. Permeability of the barrier was assessed using the fluorescent tracer FITC-dextran. We observed increased permeability of the tracer upon cocaine treatment suggesting a breach in the barrier. Then, we measured the catalytic activity of MMP-2 and MMP-9 in these cells upon cocaine exposure, since these enzymes play a key role in BBB integrity^31–33^. We detected increased activity of MMP-2 and MMP-9 in HBMECs indicating the involvement of ECM degradation in cocaine-mediated BBB breach. Inhibition of MMP activity reduced FITC-dextran permeability. Interestingly, cocaine treatment also increased prolidase expression and activity in these cells. Knock-down of prolidase abrogated permeability through the barrier suggesting a link between BBB breach and prolidase. To decipher the mechanism by which cocaine regulates prolidase, we focused on inducible nitric oxide synthase (iNOS) mediated phosphorylation of prolidase since cocaine exposure did not alter prolidase transcription. Cocaine treatment resulted in a dose-dependent increase in the phosphorylation of prolidase and iNOS expression. Inhibition of iNOS with S-methylisothiourea (SMT) decreased prolidase phosphorylation. Finally, to test a role of prolidase in transmigration through BBB, we measured monocyte transmigration through the endothelial barrier. We detected increased transmigration of monocytic cells upon cocaine treatment. Knock-down of prolidase abrogated cocaine-mediated transmigration of these cells. Collectively, these results suggest a novel mechanism involving prolidase in cocaine-mediated breach in human brain endothelial barrier.

## Results

### Cocaine treatment increases permeability of human brain endothelial cell barrier

**Figure 1:**
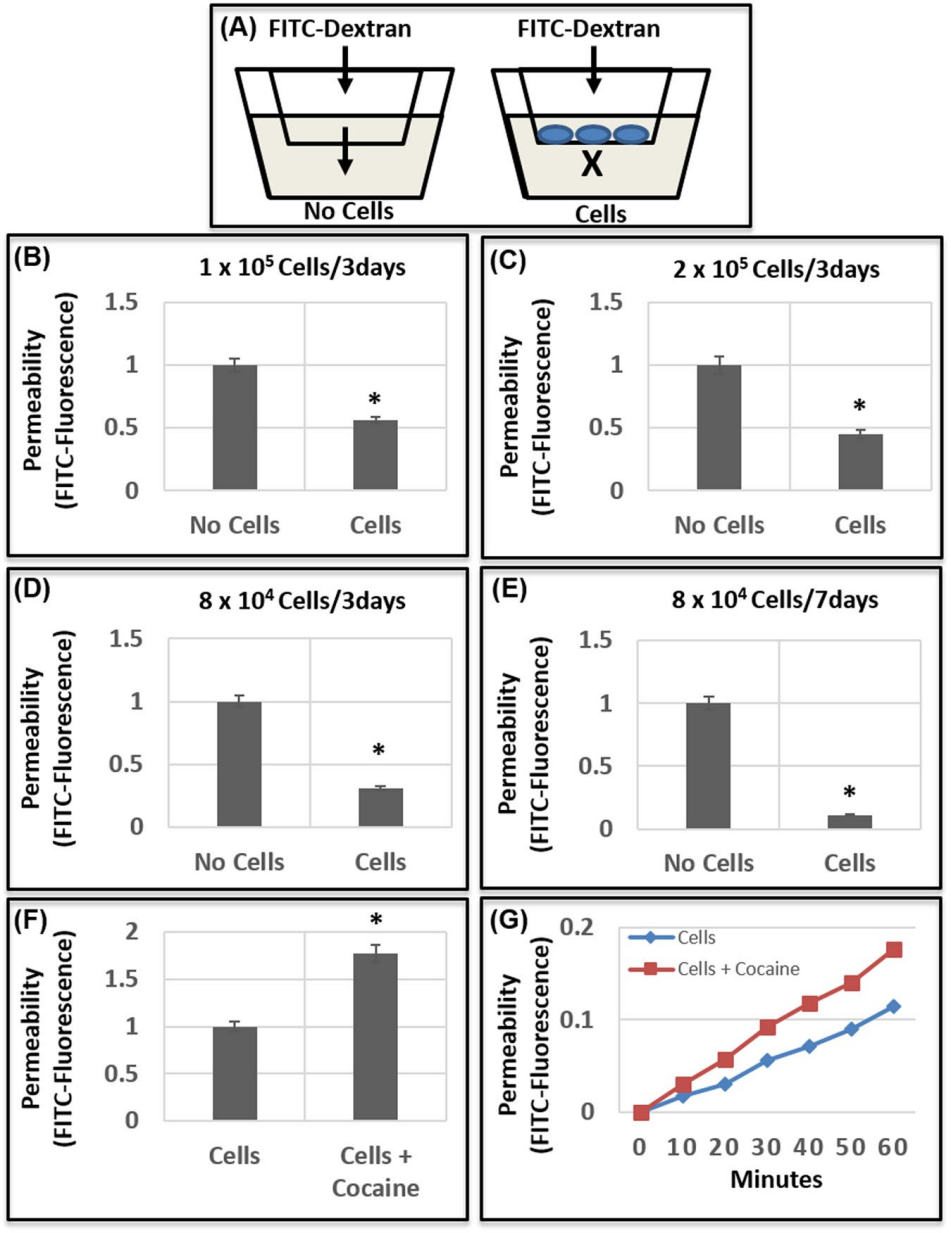
Cocaine increases permeability of endothelial barrier. (**A**) Schematic representation of the endothelial barrier model used in the study. Specified number of HBMECs [1 × 10^5^ **(B)**, 2 × 10^5^ **(C**), 8 × 10^4^ **(C**,**D)**], were seeded on transwell inserts for 3 days **(B**–**D)** and 7 days **(E)**. Establishment of a continuous layer was monitored by light microscopy. To measure permeability of the barrier, FITC-dextran was added to the upper chamber and fluorescence in the culture media of the lower chamber was measured. Transwell inserts without cells are used as the control for measuring relative permeability of the FITC-dextran. **(F**,**G)** Effect of cocaine treatment on permeability of HBMEC barrier. **(F)** 8 × 10^4^ HBMECs were seeded on a transwell for 7 days. Thereafter, the cell layer was treated with cocaine (50 μM) for 18–24 h and FITC- dextran permeability was measured. **(G)** Effects of cocaine treatment on endothelial cell permeability was measured as a function of time. Data represents the mean ± SEM of at least three determinations. *P < 0.05 represents statistical comparisons of no cells and cells in Panels (B–E), and untreated and cocaine-treated samples in Panel (F).

Cocaine exposure has been reported to compromise the integrity of the BBB^17–19^. However, the mechanism by which cocaine breaches the BBB remains largely unclear. To probe the effects of cocaine on BBB integrity, we employed an *in vitro* BBB model composed of a monolayer of human brain microvascular endothelial cells (HBMECs, Fig. 1A). HBMECs have been widely used to study the molecular and cellular details of BBB because they are a key component of the BBB and possess unique features that are distinct from the peripheral endothelial cells^51,52^. To establish an optimized HBMEC based barrier model, we seeded varied number of HBMECs on collagen-coated transwell inserts for 3 days and 7 days. Thereafter, FITC-dextran was added to the upper compartment and permeability of this tracer through the HBMEC monolayer was measured in the lower compartment by FITC fluorescence (Fig. 1A). Our results show that when 10^5^ HBMECs were seeded for 3 days, the barrier formed by the monolayer reduced the permeability of FITC-dextran to the lower chamber by ~ 50% compared to the inserts containing no cells (Fig. 1B). Increasing the number of cells to 2 × 10^5^ and establishing the monolayer for 3 days resulted in a ~60% decrease in FITC-dextran permeability (Fig. 1C). Interestingly, a marked reduction (>70%) in the permeability was observed in a monolayer established with reduced number of cells (8 × 10^4^) cultured for 3 days (Fig. 1D). Accordingly, after 7 days, the barrier of this monolayer resulted in a >90% decrease in FITC-dextran permeability (Fig. 1E). Using the conditions of Fig. 1D, we tested the effects of cocaine treatment on BBB integrity. The HBMEC barrier was treated with cocaine (50 μM) overnight and then permeability of FITC-dextran was measured. Data in Fig. 1F show that cocaine treatment resulted in an increased permeability (up to 2-fold) of the tracer through the barrier. Additionally, time dependent studies showed that upon cocaine treatment optimal permeability of FITC-dextran to the lower chamber is observed after 60 min (Fig. 1G). Collectively, these results indicate that cocaine treatment enhances permeability of the barrier formed by HBMEC monolayer, akin to a breach of BBB.

### Cocaine exposure activates matrix metalloproteinases in human brain endothelial cells

**Figure 2:**
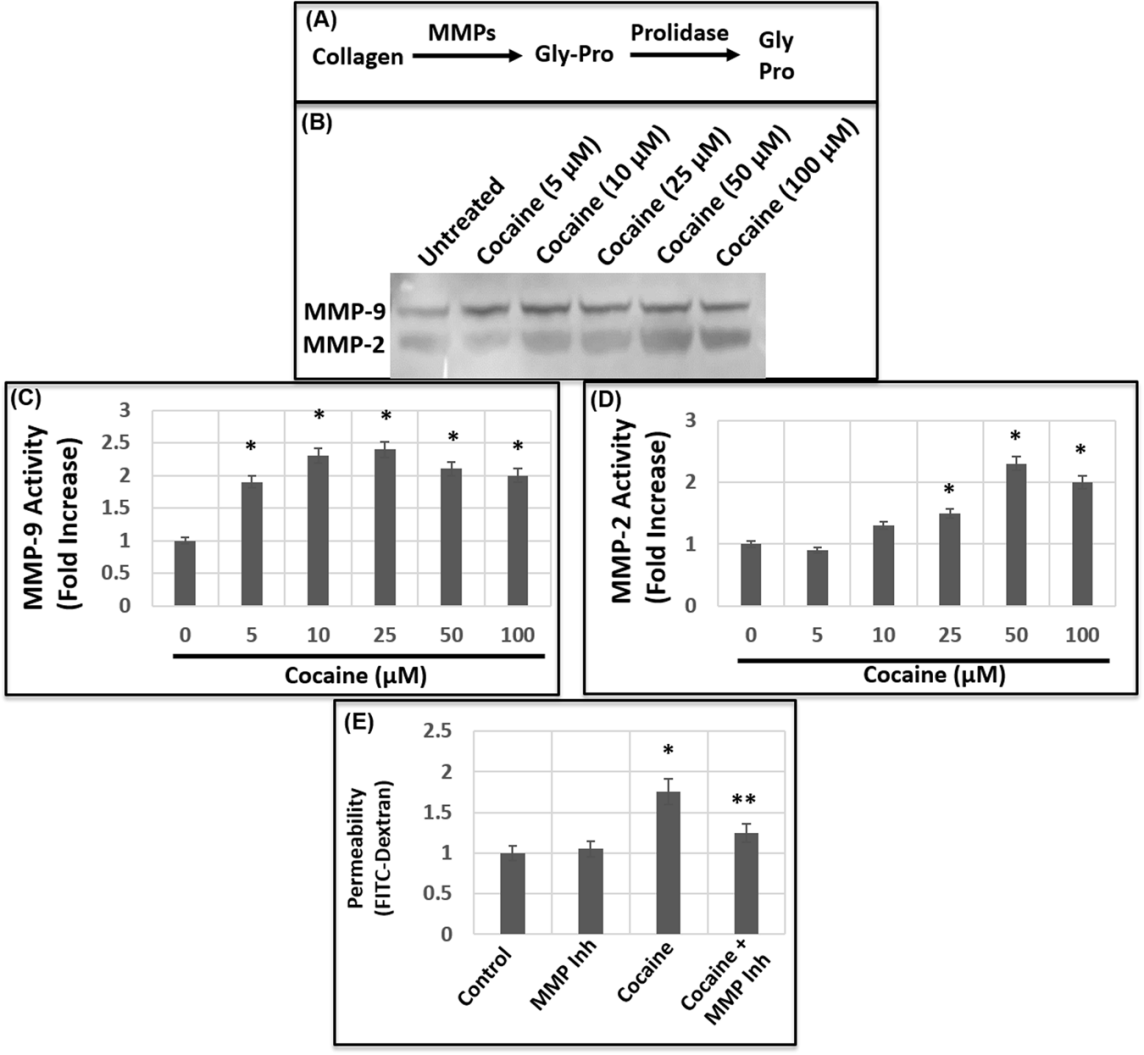
Cocaine treatment activates MMP-9 and MMP-2. (**A**) Schematic representation of the steps and enzymes involved the biochemical degradation of ECM-associated collagen. **(B)** HBMECs were treated with a range of concentrations of cocaine for 18–24 h and the culture supernatants were used as the source of MMP-9 and MMP-2 activity by zymography. **(C**,**D)** Densitometry analysis of MMP-9 **(C)** and MMP-2 **(D)** activity in HBMECs from three independent experiments. **(E)** Effects of MMP inhibition on cocaine-mediated increased permeability. The HMBEC monolayer in the transwell were treated with cocaine (50 μM) or cocaine + the MMP inhibitor GM6011. Then FITC-permeability was measured. Data represents the mean ± SEM of at least three determinations. *P < 0.05 represents statistical comparison of untreated and cocaine-treated samples, whereas that of cocaine-treated sample to cocaine and GM6011-treated samples by **P < 0.05.

In the CNS, MMP-2 and MMP-9 are critical for the maintenance and disruption of BBB integrity^34,35^. Therefore, to gain insight into the mechanism by which cocaine enhances BBB permeability, we measured the catalytic activity of MMP-2 and MMP-9 in HBMECs using gelatin-zymography assay. Cells were grown in collagen-coated plates and treated with cocaine in a dose-dependent manner (1–100 μM). These concentrations were used to reflect the range of cocaine levels with physiological relevance^53–58^. 24 h post-treatment, the culture supernatants were used as the source of MMP-2 and MMP-9 activity. Our results revealed that cocaine treatment resulted in the induction of both MMP-9 and MMP-2 activity (Fig. 2B–D). MMP-9 activity was induced in cells treated with 5 μM of cocaine followed by a steady increase with higher concentration of cocaine treatment. A maximum induction of MMP-9 activity (up to 2–3 fold) was observed in cells treated with 25 μM of cocaine (Fig. 2C) that remained relatively steady with 50 μM and 100 μM of cocaine. Similarly, cocaine treatment also induced MMP-2 activity in HBMECs. Interestingly, the activity of MMP-2 showed minimal induction with 5 μM and 10 μM of cocaine (Fig. 2D), even though MMP-9 activity was induced at these concentrations. Cells treated with 25 μM of cocaine showed increased MMP-2 activity with optimal induction observed with 50 μM of cocaine treatment (Fig. 2D). Collectively, these results provide biochemical evidence of cocaine-induced MMP activation in HBMECs.

### Inhibition of MMP abrogates cocaine-induced permeability of the barrier

Our results showed that cocaine treatment increases the permeability of HBMEC barrier (Fig. 1) concurrent with the activation of MMP-9 and MMP-2 (Fig. 2). Therefore, we tested whether MMP activation by cocaine is linked to the breach of the endothelial cell barrier. We carried out FITC-dextran permeability assay in the presence of a pharmacologic inhibitor GM6011 that potently inhibits MMP activity^59^. Addition of GM6011 alone to the HBMEC monolayer showed minimal effect on FITC-dextran permeability (Fig. 2E), establishing the utility of this reagent to study effects of MMPs on endothelial cell barrier. As expected, cocaine treatment increased the permeability of the FITC-dextran through the barrier when compared to the untreated cells (Fig. 2E). Interestingly, cocaine-induced permeability was reduced in the presence of the MMP inhibitor (Fig. 2E). These results indicate that the breach in the endothelial cell barrier by cocaine is mediated in part by the catalytic activity of MMPs.

### Cocaine treatment induces the expression and activity of prolidase

**Figure 3:**
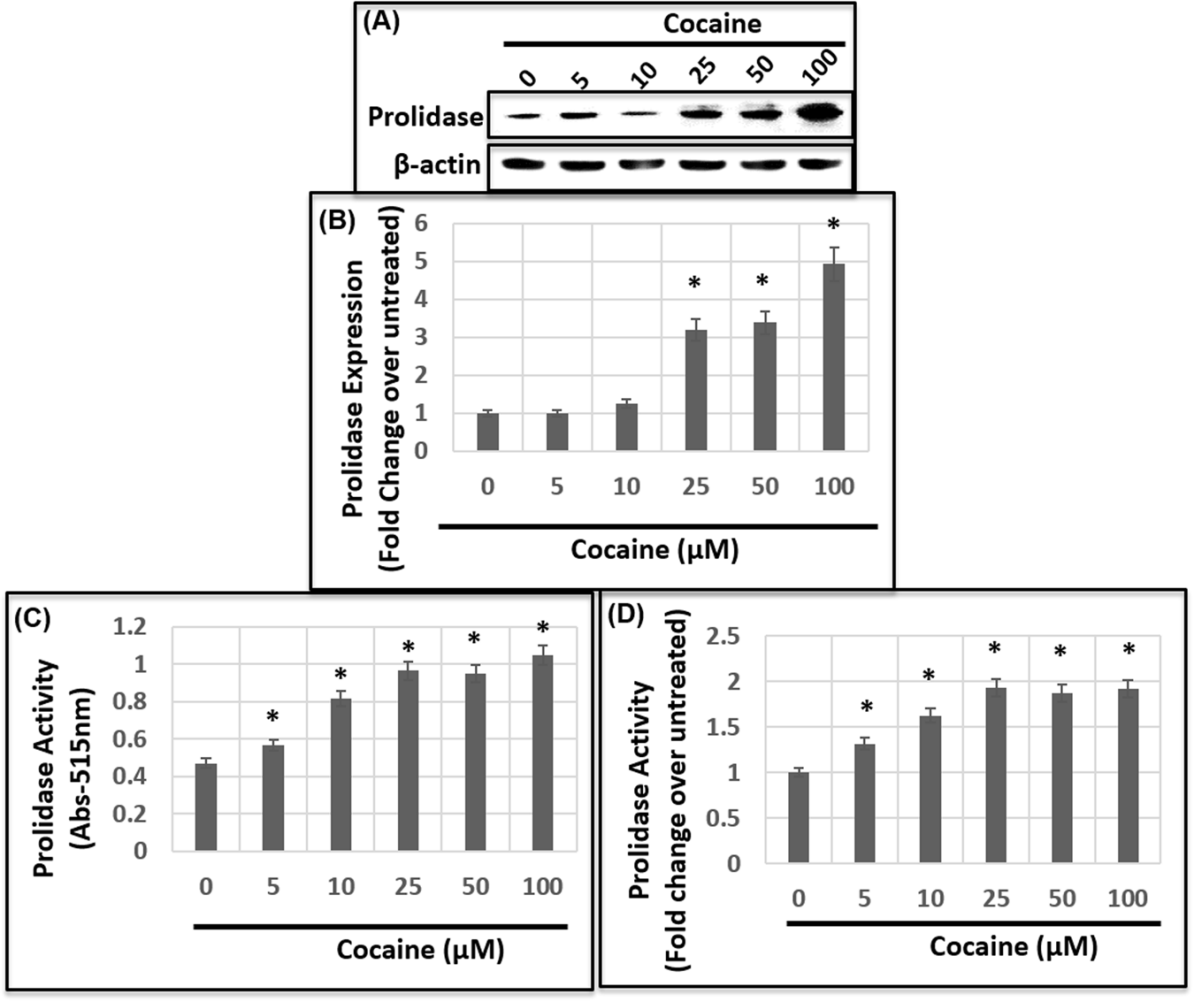
HBMECs were treated with a range of concentrations of cocaine for 18–24 h and the cellular lysates were used to measure prolidase expression and activity. **(A)** Immunoblot analysis of prolidase expression. Equal amounts of protein in the cellular lysates were analyzed by denaturing PAGE and probed by anti-prolidase and anti-GAPDH antibodies. **(B)** Densitometry analysis of prolidase expression relative to GAPDH from three independent experiments. **(C**,**D)** Effects of cocaine treatment on prolidase activity. Prolidase activity in the cellular lysates was measured by a colorimetric method that measures amounts of proline released. **(C)** Prolidase activity data as measured by absorption at 515 nm. **(D)** Relative prolidase activity data plotted as fold change over control untreated cells. Data represents the mean ± SEM of at least three determinations. *P < 0.05 represents statistical comparison of untreated and cocaine-treated samples.

Our results depicted in Fig. 2E showed that inhibition of MMP activity can partially abrogate cocaine-induced permeability of the HBMEC barrier. Accordingly, MMPs are known to initiate the breakdown of the ECM associated collagen during BBB breach^31–33^. However, the final step of collagen degradation in this process require the catalytic activity of prolidase (Fig. 2A)^49,50^. Therefore, we tested the effects of cocaine on induction of prolidase in HBMECs. Cells were treated with increasing concentration of cocaine (1–100 μM) and the cellular lysates were analyzed for prolidase expression and catalytic activity. Immunoblot analysis revealed a dose-dependent increase in prolidase expression in cocaine-treated cells when compared to the untreated cells (Fig. 3A). Treatment with 5 μM and 10 μM of cocaine showed a minimal change in prolidase expression, whereas with 25 μM cocaine treatment, expression of prolidase was markedly increased up to 3-fold (Fig. 3B). A maximum increase of ~ 5-fold in prolidase expression was achieved in cells treated with 100 μM cocaine (Fig. 3B). These results establish that cocaine treatment above 25 μM concentration increases prolidase expression in HBMECs.

Subsequently, we examined whether cocaine treatment resulted in an increased catalytic activity of prolidase. We employed a colorimetric assay that measures the amount of proline released by the catalytic activity of prolidase upon hydrolysis of the dipeptidic substrate glycine-proline (Gly-Pro)^60^. Lysates of HBMECs treated with increasing concentrations of cocaine were incubated with MnCl_2_ to activate prolidase. The activated lysates were then used as the source of prolidase activity. Quantification of the released proline show a dose dependent increase in prolidase activity in HBMECs treated with cocaine (Fig. 3C,D). The increase in activity was observed in cells treated with 5 μM cocaine. The activity steadily increased to an optimum with 25 μM cocaine and reaching saturating levels with 50 μM and 100 μM cocaine. These data show that cocaine treatment enhances prolidase expression and catalytic activity in HBMECs.

### Prolidase regulates cocaine-induced breach of the endothelial cell barrier

**Figure 4:**
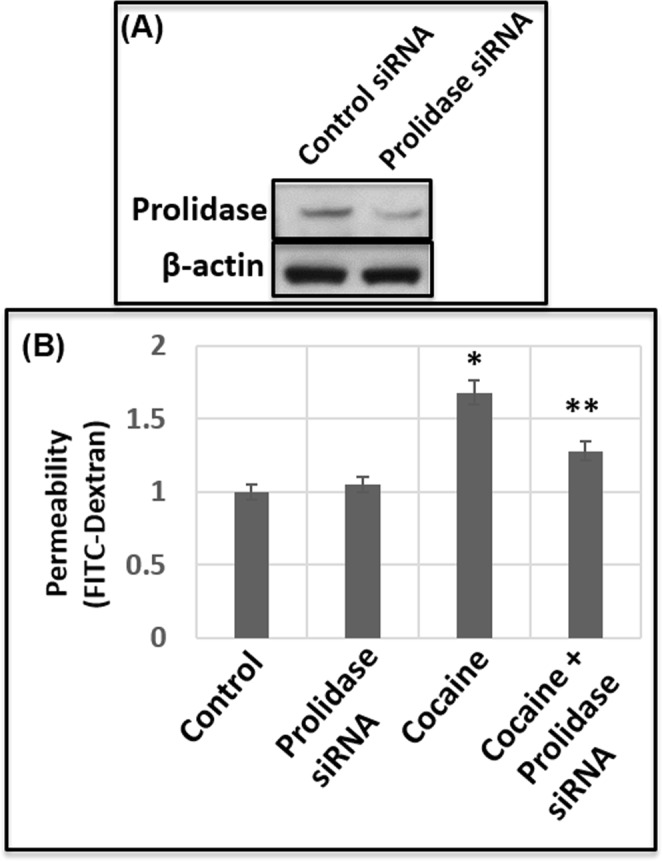
Prolidase regulates cocaine-induced permeability through endothelial cell barrier. (**A**) Prolidase expression was knocked-down by siRNA-based method and confirmed by immunoblot analysis. **(B)** Cocaine (50 μM) was added to the HMBEC monolayer in the trans-well without or with prolidase knock-down conditions. Then FITC-permeability in these trans-wells was measured. Data represents the mean ± SEM of at least three determinations. *P < 0.05 represents statistical comparison of untreated and cocaine-treated samples, whereas that of cocaine-treated sample with and without prolidase knock-down is depicted by **P < 0.05.

Since prolidase plays critical role in ECM degradation^49,50^, we tested the role of prolidase in cocaine-induced breach of the endothelial cell barrier. We employed a catalytic inhibitor of prolidase (CBZ-Pro) in our HBMEC monolayer based barrier model. Unfortunately, treatment of CBZ-Pro showed high-level of toxicity towards HBMECs (data not shown). Therefore, probing the role of prolidase using this inhibitor was not feasible in our transwell assay. As an alternative, we employed siRNA based prolidase knock-down approach. Data in Fig. 4A show that transfection of prolidase specific siRNAs into HBMECs resulted in a dramatic reduction in prolidase expression. Using these knock-down conditions, we conducted permeability assay in the barrier model. Data in Fig. 4B reveal that knock-down of prolidase in untreated HBMECs minimally affected FITC-dextran permeability through the barrier. Interestingly, prolidase knock-down resulted in a marked reduction in FITC-dextran permeability in cocaine treated cells. These results suggest that the breach in the endothelial cell barrier by cocaine depends in part on prolidase.

### Cocaine-induced transmigration of monocytes is regulated by both MMPs and Prolidase

**Figure 5:**
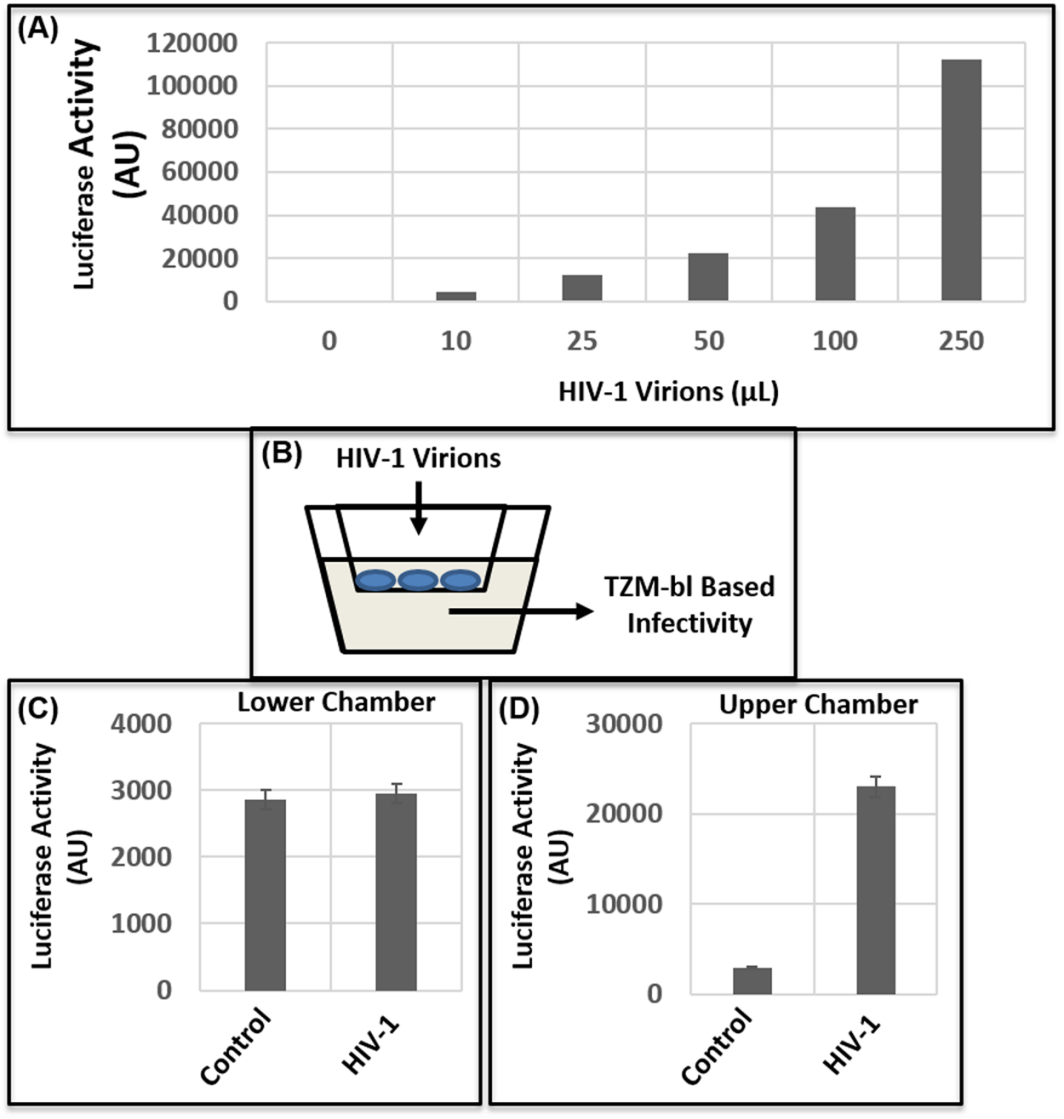
Effect of cocaine on transmigration of HIV-1 particles. (**A**) Cell-free virions were used for transmigration assays. Infectivity of these virions were measured by TZM-bl based luciferase reporter assay. **(B)** Schematic representation of transmigration assay of HIV-1 particles through the endothelial cell barrier. To measure transmigration of HIV-1 particles, 8 × 10^4^ HBMECs were seeded on a transwell for 7 days. Thereafter, virions (200 μL) were added to the upper chamber and transmigration was measured by using the media in the lower chamber in TZM-bl based infectivity assay. **(C)** Transmigration of HIV-1 particles after 24 h as measured by infectivity. **(D)** Infectivity of virions added to the upper chamber. Data represents the mean ± SEM of at least three independent determinations.

**Figure 6:**
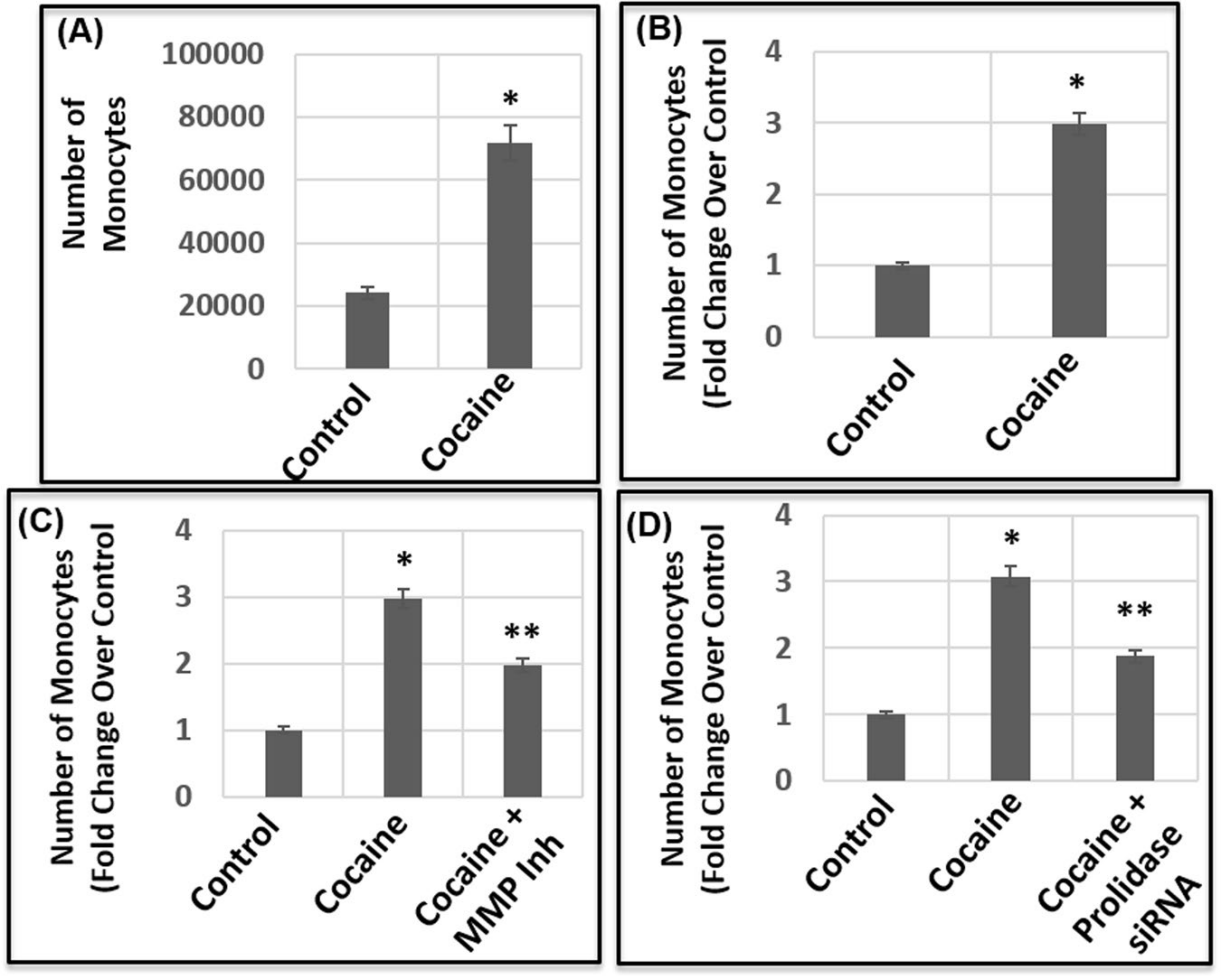
Effect of cocaine on transmigration of monocytic cells across endothelial cell barrier. THP1 monocytic cells were added to the upper chamber of a HBMEC monolayer established on a trans-well insert and transmigration of cells was measured in untreated and cocaine-treated (50 μM) samples by counting cells in the lower chamber. **(A)** Transmigration data as measured by number of THP1 cells in the lower chamber. **(B)** Relative transmigration data plotted as fold change over control untreated cells. **(C)** Effects of MMP inhibition on the transmigration of THP-1 monocytes. The HBMEC monolayer was treated with cocaine or cocaine + GM6011 followed by addition of THP-1 cells. **(D)** Effects of Prolidase on cocaine-induced transmigration of THP-1 monocytes. Prolidase expressing and prolidase knock-down HBMEC monolayer was treated with cocaine (50 μM) followed by addition of THP-1 cells. Data represents the mean ± SEM of at least three determinations. *P < 0.05 represents statistical comparison of untreated and cocaine-treated samples, whereas that of cocaine-treated sample with and without MMP inhibitor in Panel (C) and prolidase knock-down in Panel (D) are depicted by **P < 0.05.

Our results described in Fig. 1 through Fig. 4 indicate that cocaine-mediated breach in the barrier of HBMECs is regulated by MMPs and Prolidase. Interestingly, BBB breach by cocaine can enhance HIV-1 neuro-invasion via increased transmigration of the virus particles and infected cells. Therefore, we tested transmigration of infectious HIV-1 particles through the barrier of HBMEC monolayer. Cell-free virions were generated by activating chronically infected ACH-2 cells and collecting the culture supernatants^61^. The virus particles were centrifuged and filtered prior to inoculation into the upper chamber of the barrier^61^. Transmigration of the virions was determined by measuring infectivity of the supernatant in the lower chamber. Surprisingly, we observed minimal infectivity in the media in the lower chamber suggesting lack of virus transmigration through the barrier (Fig. 5). Thereafter, we measured transmigration of THP1 monocytic cells through the barrier of HBMEC monolayer in the presence of cocaine. Transmigration was quantified by adding THP1 cells to the upper chamber of the transwell and counting the number of cells transmigrated to the lower chamber in a time-dependent manner. Data from these assays revealed that number of THP1 cells transmigrating to the lower chamber increased as a function of time with maximum number of cells transmigrating after 24 h (Fig. 6). Treatment of the HBMEC barrier with cocaine (25 μM) revealed an increased transmigration of monocytic cells (Fig. 6A,B). Thereafter, we tested whether cocaine-induced transmigration is dependent on MMPs and prolidase. Interestingly, pretreatment with a MMP inhibitor abrogated cocaine-induced transmigration of THP1 cells (Fig. 6C). Similarly, knock-down of prolidase also resulted in a decreased transmigration in cocaine-treated cells (Fig. 6D). These results suggest that MMPs and prolidase play an important role in cocaine-induced increased transmigration of monocytic cells through endothelial cell barrier.

### Cocaine-induced activation of prolidase is regulated by serine/threonine phosphorylation

**Figure 7:**
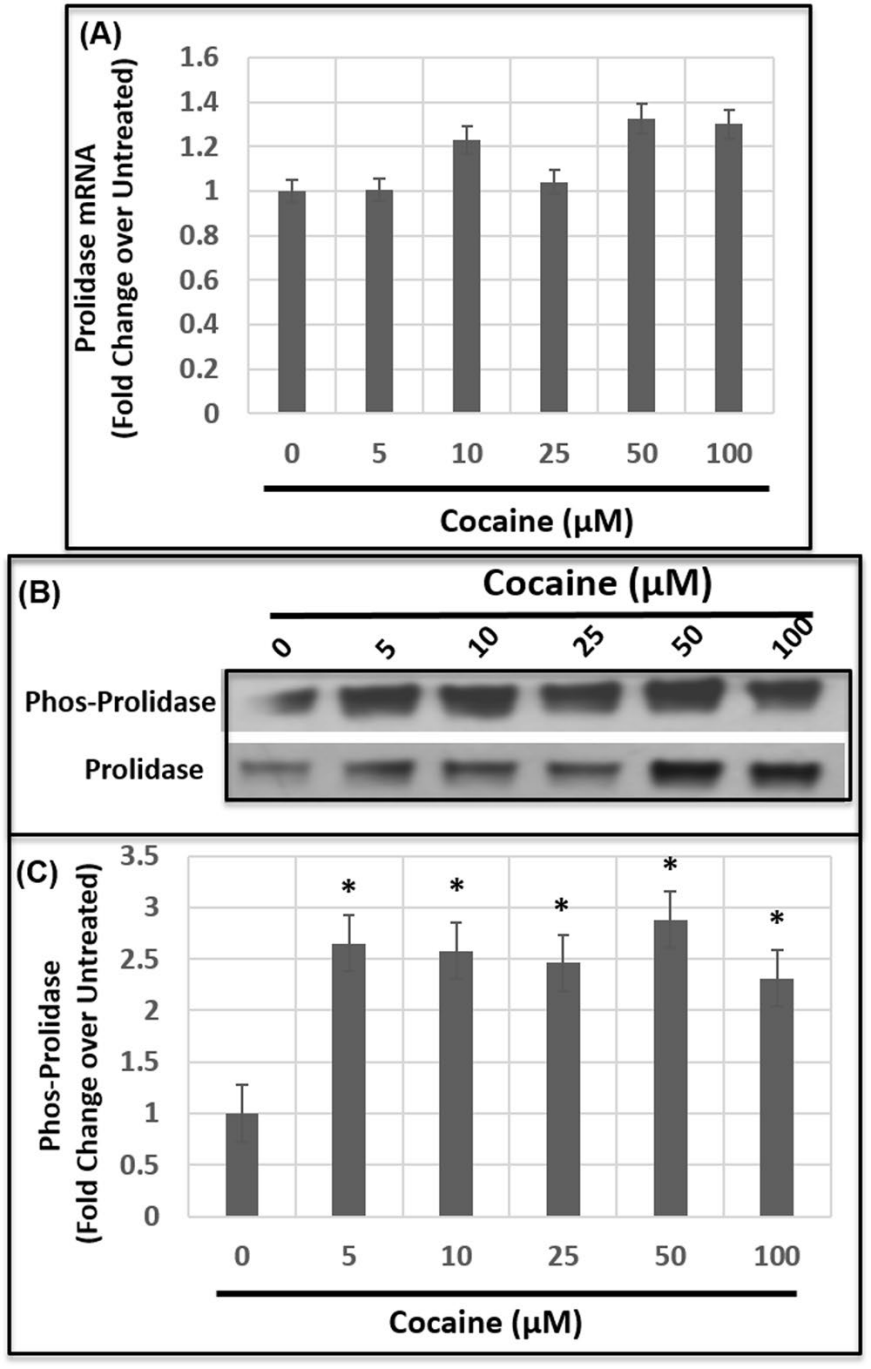
Cocaine treatment induces Ser/Thr phosphorylation of prolidase. HBMECs were treated with cocaine overnight and the cellular lysates were used to measure mRNA levels of prolidase, and serine/threonine phosphorylated prolidase. **(A)** Quantification of prolidase mRNA levels. Total RNA from untreated and cocaine-treated cells was subjected to qPCR and prolidase mRNA levels were normalized to the levels of GAPDH mRNA. Prolidase mRNA in cocaine-treated cells were expressed as fold changes over the untreated control cells. **(B)** Immunoblot analysis of phospho-prolidase expression. To determine levels of Ser/Thr phosphorylation, cellular lysates were immunoprecipitated with anti-prolidase antibody and analyzed by immunoblot using anti-phospho Ser/Thr antibody or with anti-prolidase antibody. **(C)** Densitometry analysis of phospho-prolidase levels relative to total prolidase from three independent experiments. Data represents the mean ± SEM of at least three determinations. *P < 0.05 represents statistical comparison of untreated and cocaine-treated samples.

To understand the mechanism by which cocaine regulates prolidase, first we measured the mRNA levels of prolidase in HBMECs. RNA isolated from these cells were subjected to quantitative PCR (qPCR). Results depicted in Fig. 7A indicate that cocaine treatment in a dose dependent manner minimally altered the mRNA levels of prolidase. Surprisingly, the mRNA levels prolidase did not increase in cells treated with 25 μM-100 μM of cocaine, although a 3–5 fold increase in prolidase protein expression was observed in these cells (Fig. 3A). These results suggested that cocaine treatment does not alter prolidase transcription. Interestingly, comparing the prolidase expression (Fig. 3B) to the activity (Fig. 3C,D) revealed that in cells treated with 5 μM and 10 μM of cocaine, prolidase activity was markedly elevated without any increase in expression. These observations suggest the involvement of post-transcriptional/post-translational mechanisms for cocaine-induced activation of prolidase.

There is evidence that prolidase activity is regulated by phosphorylation of prolidase^60^. Specifically phosphorylation of the serine/threonine residues in the prolidase protein has been shown to affect the catalytic activity^60^. Therefore, we tested whether cocaine treatment affected phosphorylation of prolidase in HBMECs. Cellular lysates of untreated and cocaine treated cells were incubated with prolidase specific antibody for immunoprecipitation. The immunoprecipitated prolidase protein was subjected to western blot and then probed for with anti-phospho-serine/threonine antibody. Data from these analysis revealed that cocaine treatment markedly enhanced prolidase phosphorylation (Fig. 7B). Interestingly, treatment of cells with 5 μM and 10 μM of cocaine showed substantially increased phosphorylation of prolidase (Fig. 7C). The phosphorylation levels correlated with the increase in prolidase activity in cells treated with 5 μM and 10 μM of cocaine (Fig. 3A,B). These results suggested a link between prolidase phosphorylation and catalytic activity in cocaine treated cells.

### Cocaine upregulates Inducible Nitric Oxide Synthase (iNOS) for Prolidase phosphorylation

**Figure 8:**
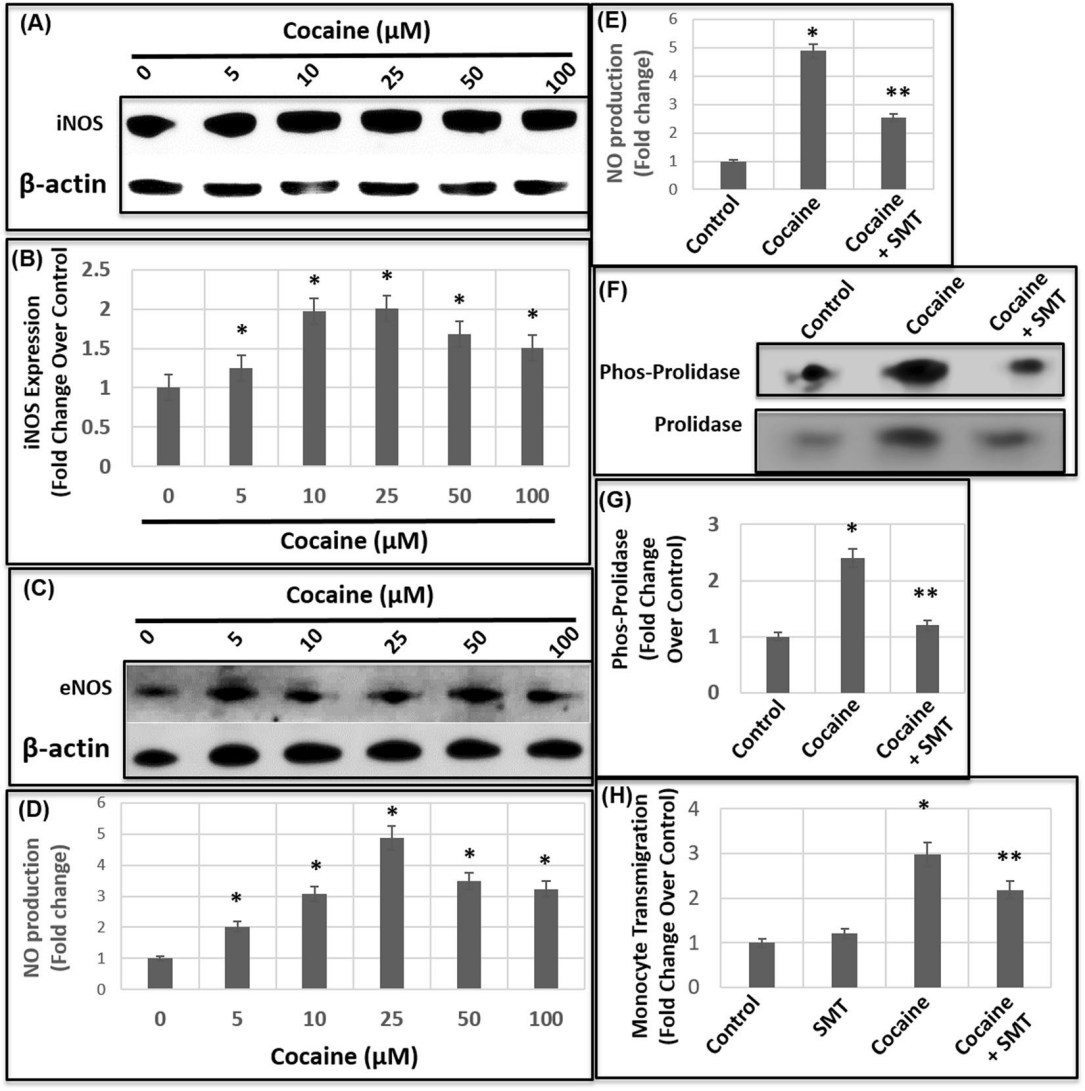
Cocaine induces iNOS to regulate prolidase phosphorylation. (**A**) Cellular lysates of untreated and cocaine-treated HBMECs were analyzed for iNOS expression by immunoblot. **(B)** Densitometry analysis of iNOS expression relative to actin from three independent experiments. Immunoblot analysis of phospho-prolidase expression. **(C**) eNOS expression in the cellular lysates of untreated and cocaine-treated HBMECs. **(D)** Measurement of NO levels in the culture supernatant of HBMECs treated without or with increasing concentrations of cocaine. **(E)** Effects of iNOS inhibition on NO production. HBMECs were treated with cocaine (25 μM) or iNOS inhibitor (SMT) followed by measurement of NO levels in the culture supernatant. **(F)** Effects of iNOS inhibition on phosphorylation of prolidase. HBMEC monolayer was treated with cocaine (50 μM) or iNOS inhibitor (SMT) followed by measurement of phospho-prolidase levels. **(G)** Densitometry analysis of iNOS inhibition on phospho-prolidase levels relative to total prolidase from three independent experiments. **(H)** Effects of iNOS inhibition on cocaine-induced transmigration of THP-1 monocytes. HBMEC monolayer was treated with cocaine (50 μM) and/or iNOS inhibitor (SMT) followed by addition of THP-1 cells. Data represents the mean ± SEM of at least three determinations. *P < 0.05 represents statistical comparison of untreated and cocaine-treated samples in Panels (B,D,E,H), whereas **P < 0.05 depicts that of cocaine-treated sample with and without iNOS inhibitor in Panels (E,G,H).

To better understand the mechanism by which cocaine treatment induces prolidase phosphorylation, we focused on nitric oxide (NO). The rationale is two-fold; (1) NO regulates prolidase phosphorylation^60^ and (2) production of nitric oxide (NO) by Inducible Nitric oxide (iNOS) is linked to the breach of BBB^62,63^. To measure iNOS induction, HBMECs were treated with cocaine at increasing concentrations for 24 h and cell lysates were analyzed by immunoblot. As described in Fig. 8A, a marked increase in iNOS expression was observed in cocaine-treated cells as compared to the untreated cells. Densitometry analysis illustrated a dose-dependent increase in iNOS levels starting at 5 μM cocaine and increasing up to two-fold with 10 μM of cocaine. There was no further increase in phosphorylation of prolidase with 25 μM cocaine treatment (Fig. 8B). These results highlight that the levels of iNOS in cells treated with 5 μM and 10 μM of cocaine correlate with the increase in prolidase phosphorylation (Fig. 7B,C) and increase in prolidase activity (Fig. 3D).

In addition to iNOS, endothelial cells possess another form of NOS- Constitutive or endothelial NOS (cNOS or eNOS)^64^. Given that both iNOS and eNOS can produce NO, we also measured the expression of eNOS in HBMECs treated with and without cocaine. Immunoblot analysis of cellular lysates revealed that the levels of eNOS were not significantly altered in cells treated with cocaine (Fig. 8C). Even at the highest concentration of cocaine used (100 μM), the level of eNOS was minimally increased when compared to the control cells (Fig. 8C). These results are in contrast to the marked increase in iNOS expression in cocaine-treated cells (Fig. 8A,B), suggesting that cocaine treatment preferentially upregulates iNOS in HBMECs.

Next, we measured the amount of NO in cocaine treated cells, since NO produced by iNOS has been shown to mediate Prolidase phosphorylation^60^. We measured NO levels in the supernatants of the HBMCEs after cocaine treatment by using a modified Griess method^65^. In this method, we measured nitrites and nitrates in the culture supernatants, since NO is highly unstable and degrades rapidly to these metabolites^65^. The amount of NO was determined through the extrapolation of the data to a standard curve generated using KNO_3_. Results from these analyses reveal that cocaine treatment resulted in a dose dependent increase in NO levels (Fig. 8C). Treatment of HBMECs with 5 μM cocaine resulted in a 2-fold increase in the NO levels compared to the untreated cells. Further increase in NO levels was observed with increasing concentrations of cocaine with a maximum of 5-fold increase observed with 25 μM cocaine treatment (Fig. 8C). These results mirror the increasing levels of iNOS in cells treated with cocaine (Fig. 8B) suggesting a correlation between iNOS expression and NO production.

Finally, to determine that iNOS is mediating the phosphorylation of prolidase, we treated HBMECs with a selective iNOS inhibitor SMT. Treatment of SMT showed dramatic reduction in NO levels in cocaine treated cells (Fig. 8E) demonstrating efficacy of the iNOS inhibitor to reduce NO production. As shown in Fig. 8F,G, prolidase phosphorylation was abrogated in the presence of SMT establishing that the phosphorylation of prolidase is mediated by iNOS. Finally, inhibition of iNOS also abrogated cocaine-induced transmigration of THP-1 monocytes (Fig. 8H), establishing that prolidase phosphorylation plays an important role in cocaine-mediated transmigration of monocytes. Given that phosphorylation regulates prolidase activity^60^, these studies also suggest that the enzymatic activity of prolidase is important for transmigration across the endothelial cell barrier.

## Discussion

The BBB formed by a continuous layer of tightly linked microvascular endothelial cells is supported by the tight junction proteins and the basement membrane^20–22^. This unique structural network enables the BBB to be highly selective for the transport of specific molecules to the brain parenchyma^20–22^. It is noteworthy that the functional and structural integrity of the barrier is challenged by a number of internal and external assaults including drug use^19^. Cocaine is a highly addictive psychostimulant drug that adversely affects CNS function^66–68^ and causes breach in the BBB^17,26,69^. Our results demonstrating that cocaine treatment enhances permeability of a human brain endothelial cell barrier (Fig. 1), support cocaine-mediated BBB breach. Even though the precise mechanism underlying cocaine-mediated BBB breach remains poorly understood, it has been reported that cocaine alters expression of tight junction proteins of the barrier endothelium^17,19,70^. Additionally, cocaine exposure has been suggested to elevate the levels of pro-inflammatory cytokines and chemokines^71–73^. Thus, alterations in tight junctions and an inflammatory response has been implied as the possible mechanism(s) driving cocaine-mediated BBB breach.

In this study, we provide compelling evidence for a novel mechanism mediated by the ECM degrading enzymes MMP and Prolidase in cocaine-mediated breach of the BBB. The ECM composed of type IV collagen, fibronectin, laminin, and various proteoglycans, provides structural and functional support to the cellular components of the BBB^28,29,32^. Given the dynamic nature of the BBB to continuously separate the brain parenchyma from the periphery, the ECM plays a key role in maintaining the integrity of the barrier^28,29,32^. While a number of studies have examined cocaine’s effect on the cellular components of the BBB^17–19,24^, very little is known about the effects of cocaine on the ECM. There is circumstantial evidence that cocaine addiction and relapse is associated with reorganization of the ECM and activation of MMPs that initiate degradation of ECM-associated collagen^46–48^. Cocaine treatment has also been shown to increase transcription of the MT-MMP-1^48^ even though the functional relevance of this study remains largely unclear since MMP catalytic activity is required for ECM degradation. The MMPs are secreted as zymogens and cleaved to their active form in the extracellular milieu^31–33^. A specialized group of MMPs called gelatinases (also known as type IV collagenases) are widely expressed in the CNS by almost all cell types^74^. Gelatinase A (MMP-2) and gelatinase B (MMP-9) specifically digest type IV collagen of the basement membrane^75^. Our studies show that in HBMECs, cocaine treatment induces the catalytic activity of both MMP-2 and MMP-9 (Fig. 2) supporting a role of these MMPs in BBB permeability. Accordingly, inhibition of MMP activity abrogated cocaine-mediated enhanced permeability through the endothelial cell barrier (Fig. 2), demonstrating a functional link between collagen degradation and cocaine-mediated BBB breach.

Collagen being the major component of the ECM is essential for the integrity of tissue architecture^28,29,32^. The collagens have a triple-stranded helical structure with a high content of proline and hydroxyproline (~25–30%)^76^. The breakdown of collagen in the basement membrane by MMPs release imido-dipeptides and imido-tripeptides containing C-terminal proline or hydroxyproline (Fig. 2A)^49,50^. It is noteworthy that these peptides are specific substrates of prolidase-a specialized manganese dependent exopeptidase^49,50^. Therefore, collagen metabolism and ECM remodeling are dependent and regulated by the catalytic activity of prolidase^49,50^. Indeed, mutation in this enzyme causes prolidase deficiency- an autosomal disorder associated with defective collagen metabolism that affects connective tissues of a variety of organs^77^. However, the role of prolidase in cocaine-mediated collagen breakdown and BBB disruption has not been previously described. Our results demonstrate that cocaine treatment enhances both expression and activity of prolidase in HBMECs (Fig. 3). Furthermore, our observations establish that reducing prolidase expression abrogates cocaine-mediated enhanced permeability (Fig. 4) suggesting that prolidase plays a key role in cocaine-mediated BBB breach.

Prolidase plays important roles in collagen metabolism and matrix remodeling of the BBB^49,50^. Therefore, it is implicated in an array of neurological disorders including Parkinson’s disease^78,79^, Schizophrenia^80,81^, Bipolar disorder^82^ and Alzheimer’s disease^83^. In addition, prolidase plays a key role in several physiological and other pathological processes such as wound healing, inflammation, angiogenesis, cell proliferation, and carcinogenesis^49,50,60,76,84,85^. Surprisingly, little is known about the regulation of prolidase (PEPD) gene expression. There is evidence that prolidase is transcriptionally regulated by hypoxia-inducible factor-1 (HIF-1)^85^. Interestingly, our results demonstrate that cocaine-mediated increase in prolidase expression is not due to increased transcription (Fig. 7A). To understand the mechanism by which cocaine regulates prolidase we considered phosphorylation since it has also been reported that prolidase activity is post-translationally regulated by serine/threonine phosphorylation^60^. Our studies showing increased phosphorylation of prolidase by cocaine exposure supported a post-translational mechanism for the activation of prolidase (Fig. 7). Importantly, phosphorylation of prolidase could stabilize the protein and may explain the increase in prolidase expression without elevated levels of mRNA.

Our results also identified that prolidase phosphorylation is dependent on the iNOS pathway given that and inhibition of iNOS reduced prolidase phosphorylation (Fig. 8C,D). This is in accordance with the published study by Surazynski *et al*., that iNOS mediated phosphorylation regulates prolidase catalytic activity. Nitric oxide (NO) generated by iNOS, is a signaling molecule that regulates multiple processes including collagen synthesis and matrix remodeling. We focused on iNOS expression because previous studies have reported that cocaine increases iNOS levels in different organs including brain^86,87^. Accordingly, our results demonstrate that cocaine treatment induced iNOS expression in HBMECs (Fig. 8A,B) without altering the expression of eNOS (Fig. 8C). These results strongly suggested the specificity of cocaine for activating iNOS mediated pathway. Moreover, inhibition of iNOS reduced cocaine-mediated enhanced permeability of the endothelial cell barrier, providing further evidence that prolidase is functionally involved in cocaine-mediated BBB breach. Collectively, our data strongly suggest that cocaine-induced iNOS stimulates the MMP-prolidase axis resulting in enhanced collagen breakdown leading to disruption in the integrity of the BBB.

Cocaine-mediated BBB breach has important consequences in several neurological diseases including HAND. Cocaine use has been shown to increase the incidence and severity of HAND^27^. Even though HIV does not infect neurons, both HIV and cocaine target the brain and cause CNS dysfunction^27^. In addition, given that cocaine can disrupt the BBB, it can facilitate transmigration of HIV-1 and/or infected cells across the BBB to exacerbate HAND^17–19^. Our studies indicate that HIV-1 particles lack the ability to transmigrate across the HBMEC barrier (Fig. 5). However, monocytic cells that support HIV-1 infection, can efficiently transmigrate across the barrier (Fig. 6) supporting the Trojan horse model of HIV-1 neuro-invasion. Accordingly, cocaine treatment increased the number of cells transmigrating across the barrier (Fig. 6A), in agreement with previous reports on increased monocyte transmigration through endothelial barriers upon cocaine treatment^18,69,88–90^. Finally, our results establish that regulating the ECM-degrading enzymes such as MMPs and prolidase (Fig. 6B,C) abrogate cocaine-treatment associated monocyte transmigration. These observations provide key insights into the role of ECM in BBB breach by cocaine and establish a novel mechanism for cocaine-mediated transmigration of monocytes that plays critical role in HAND.

## Materials and Methods

### Reagents and cells

Cocaine hydrochloride, FITC-dextran, MMP inhibitor (GM6011), prolidase inhibitor (N-Cbz-L-proline), iNOS inhibitor (S-methylthiourea [SMT]), N- (1-Naphthyl) ethylenediamine dihydrochloride (NEDD), sulfanilamide (SULF) vanadium (III) chloride (VCl3), potassium nitrate, and Prolidase substrate (Gly-Pro) were purchased from Sigma-Aldrich (St. Louis, MO, USA). Anti-Prolidase (a gift from Dr. James Phang, NCI/NIH), anti-phospho Ser/Thr and anti-iNOS antibodies were from Cell Signaling Technology (Danvers, MA, USA), and anti-eNOS was procured from the Bethyl Laboratories (Montgomery, TX, USA) and anti-GAPDH and anti-β actin antibodies were procured from Sigma-Aldrich (St. Louis, MO, USA). Human Brain Microvascular Endothelial Cells (HBMECs) were purchased from Sciencell (Carlsbad, CA). THP-1 cell line was obtained from the American Type Culture Collection (ATCC) (Manassas, VA). The TZM-bl reporter cell line was obtained from Dr. John C. Kappes, Dr. Xiaoyun Wu and Tranzyme Inc., through the NIH AIDS Reagent Program, Division of AIDS, NIAID, NIH.

### Cell culture

HBMECs were maintained in collagen-coated T75 tissue culture flasks in EBM-2 MV SingleQuot Kit Supplement and Growth Factors (LONZA, Walkersville, MD) supplemented with 10% (v/v) heat-inactivated fetal bovine serum (FBS) (Gibco, USA), 2 mM glutamine and 1% antibiotics (penicillin–streptomycin). TZM-bl cells were cultured in Dulbecco’s modified Eagle’s medium (DMEM) supplemented with 10% heat-inactivated fetal bovine serum (FBS), 2 mM glutamine, 1000 U/mL penicillin, and 100 mg/mL streptomycin. THP-1 cells were cultured in RPMI 1640 medium supplemented with 10% heat-inactivated FBS, 2 mM glutamine, 1000 U/mL penicillin, and 100 mg/mL streptomycin. All cells were cultured at 37 °C with 5% CO2.

### Barrier model

An *in vitro* model of the BBB was established by culturing HBMECs in transwell inserts. When cells were >90% confluent in the T-75 flasks, they were then transferred onto collagen-coated Transwell inserts (6.5 mm, pore size 0.4 μm; Corning, Sigma-Aldrich (St. Louis, MO, USA) in a 24-well culture dish. Varied number of cells (1 × 10^5^ or 2 × 10^5^ or 8 × 10^4^) were seeded for 3 days and 7 days. Cells were monitored under light microscope every day. Fresh medium was added to these cells prior to treatment with cocaine and/or inhibitors.

### Permeability assay

HBMECs cultured on transwell membranes were washed 3 times with HBSS (Life Technologies, USA). The fluorescent tracer, 70 kDa FITC-dextran (250 μg/ml) was added to the apical side of the Transwell insert, and samples were collected from the lower compartment after 10, 20, 30, 40, 50, and 60 min. Permeability of FITC-Dextran to the lower chamber was determined by measuring FITC fluorescence of the culture media in the lower chamber with a fluorescence plate reader (Biotek, USA). The permeability of the tracer through the barrier exposed to cocaine was calculated relative to that of untreated controls.

### Determination of MMP activity

HBMECs were seeded and cultured for required time period. These cells were treated with cocaine overnight in a dose dependent manner from 1–100 μM. These concentrations were used based on published literature that highlight their physiological relevance^53–58^. Supernatant of these HBMECs was concentrated using protein concentrators (Pierce, USA) and the protein levels in the concentrated supernatant was measured by BCA assay (Pierce, USA). MMP-2 and MMP-9 activity assays were carried out using gelatin-based gel zymography. Equal amounts of protein were diluted into 2× sample buffer (Life Technologies, USA) and electrophoresed on Novex zymogram gels (Life Technologies, USA). The gels were incubated at room temperature in 1x renaturing buffer for 30 min with gentle agitation. The renaturing buffer was decanted and replaced with 1x developing buffer at room temperature for 30 min with gentle agitation. The developing buffer was decanted and replaced with new developing buffer. The gels were incubated overnight and stained using coomassie blue SimplyBlue safe stain (Thermofisher, USA).

### Western blot analysis

HBMECs were treated with various concentrations of cocaine (1–100 μM) for 24 h, after which the cells were harvested and washed with PBS (1X). Cell lysates were prepared using standard protocol and protein concentrations in the lysates were quantified by BCA protein assay (Pierce, USA). Equal amounts of protein were electrophoresed on SDS-polyacrylamide gels and transferred to nitrocellulose membranes using a semi-dry blotter (Bio-Rad). Membranes were blocked with 5% (w/v) nonfat milk in Tris-buffered saline (TBS), pH 8.0 (Sigma, USA) and then probed with the primary antibody in blocking buffer. Subsequently the blot was incubated with a secondary antibody conjugated to horseradish peroxidase (1:2000). All blots were washed in TBS with Tween 20 (pH 8.0; Sigma) and developed using the enhanced chemiluminescence (ECL) procedure (Pierce, USA). Blots were routinely stripped by treating with Restore Plus stripping buffer (Pierce) and re-probed with anti-GAPDH or anti- β -actin monoclonal antibodies (Sigma) to serve as loading controls. Anti-rabbit antibody (Santacruz, Piscataway, NJ) was used as secondary antibody. Densitometry analyses were performed using LI-COR Image Studio version 5.2 software (LI-COR, USA). Data were normalized to levels of GAPDH or β-actin.

### Prolidase activity measurements

The catalytic activity of prolidase was determined using colorimetric determination of proline using Chinard’s reagent (25 g of ninhydrin dissolved at 70 °C in 600 ml of glacial acetic acid and 400 ml of 6 M orthophosphoric acid). HBMECs were harvested by scraping and centrifuged at 200 g for 5 min. The cell pellet was suspended in 100 μl of 50 mM HEPES, pH 7.8, containing protease inhibitor and phosphatase inhibitor and sonicated for 3 × 10 s at 4 °C. Lysates were then centrifuged (12,000 g, 30 min) at 4 °C and the supernatant was used for protein determination (BCA Protein Assay Kit, Pierce, USA) and prolidase activity assays. Activation of prolidase was initiated by incubating 25 μg of cell extract in 50 μl of 50 mM HEPES, pH 7.8 containing MnCl2 at a final concentration of 1 mM in the mixture. After incubation for 24 h at 37 °C, the prolidase activity reaction was initiated by adding 100 μl of the activated mixture to 100 μl of 94 mM Gly-Pro substrate for a final concentration of 47 mM. After additional incubation for 1 h at 37 °C, the reaction was terminated with the addition of 500 μl of 0.45 M trichloroacetic acid. Samples were centrifuged at 10,000 g for 15 min. The released proline was measured by adding 50 μl of the trichloroacetic acid supernatant to 200 μl of a 1:1 mixture of glacial acetic acid: Chinard’s reagent and incubated for 10 min at 90 °C. The amount of proline released was determined colorimetrically by measuring absorbance at 515 nm. The amount of proline was quantified by extrapolating the data to a standard curve prepared with a range of concentration of proline. Enzyme activity was reported in nanomoles of proline released per minute per milligram of protein.

### Immunoprecipitation based detection of prolidase phosphorylation

To measure phosphorylation of the Ser/Thr residues of prolidase, first prolidase was immune-precipitated using anti-prolidase antibody. Immuno-precipitation was conducted using the reagents and the method described by the manufacturer (Sigma, St. Louis, MO). Briefly, 25 μg protein equivalent of cellular lysates were pre-cleared by incubating with protein G-agarose at 4 °C for 3 h. The agarose mixture was pelleted by centrifugation and the supernatant was incubated overnight with the prolidase antibody at 4 °C. Then required amount of Protein G-agarose was added to the mixture and incubated at 4 °C for 3 h. The agarose mixture was pelleted and washed five times. The immune-precipitated proteins were resuspended in 50 μl of 1X denaturing sample buffer. Ser/Thr phosphorylation was probed by loading 20 μl of the sample onto a 10% SDS–PAGE and immune-blotting using anti-phospho Ser/Thr antibody.

### Measurement of NO levels

HBMECs seeded in culture dishes were treated with cocaine as described earlier and after 24 h, the quantity of NO produced in the supernatant was estimated. Since NO is highly unstable and degrades rapidly to nitrites and nitrates^64,65^, the detection of these NO metabolites was used to determine the NO concentration in the culture supernatants. In the reaction mixture the nitrate was first reduced to nitrites with vanadium (III) chloride (VCl_3_) and then the total nitrite was quantified by acidic Griess reaction^65^. A standard curve (0–200 μM) was generated by serial dilution of potassium nitrate (KNO_3_). Griess reagent was freshly made by mixing equal parts of 0.1% NEDD (N-(1-Naphthyl) ethylenediamine dihydrochloride in distilled water) and 2% SULF (sulphonilamide in 5% HCl). 50 μL of samples and standards (in triplicate) were added into a 96-well microtitre plate and to each well 50 μL VCl_3_ solution (0.8% VCl_3_ in 1 M HCl) was added. Followed by rapid addition of 50 µL Griess reagent. The reaction mixture was incubated at 37 °C for 60-min, and absorbance was measured at 540 nm. The concentration of total nitrate and nitrite in samples were extrapolated from the standard curve.

### Prolidase knockdown

Prolidase-specific siRNAs and non-specific scrambled controls were purchased from Santa Cruz Biotechnology (Texas, USA). HBMECs (2 × 10^5^ cells/well) grown in 6-well culture plates were transfected with 100–300 pM of prolidase-specific siRNAs or scrambled controls using Jetprime (Polypus, USA) as per the manufacturer’s protocol. Post transfection, cells were incubated for 36–48 h at 37 °C/5% CO2, washed with PBS (1X) and harvested by gentle scraping for protein isolation. Knock-down of prolidase was confirmed by immunoblot analysis.

### Real Time PCR analysis

For measuring prolidase mRNA expression, total RNA was isolated from untreated- and cocaine-treated cells using RNAesy Mini kit (Qiagen) and cDNA synthesis was carried out using iScript cDNA synthesis kit (Bio-Rad). qPCR assay was performed by subjecting 50 ng of cDNA to iTaq Universal SYBR Green chemistry (Bio-Rad, USA) using primers specific for prolidase [Forward: 5′-TCGATGTTGACACTGGGAAG -3′ and Reverse: 5′-CTCCTTGAAGTGCTCCTTGG-3′] and GAPDH [Forward: 5′-GAAGGTGAAGGTCGGAGTC-3′ and Reverse: 5′-GAAGATGGTGATGGGATTTC-3′] as per manufacturer’s instructions. The expression levels of prolidase mRNA were normalized to that of GAPDH mRNA levels. Relative expression of prolidase mRNA in untreated control and treated samples is expressed as 2-delta Ct values as described previously and fold changes are calculated by comparing the 2-delta Ct values of the treated sample with that of untreated control.

### Transmigration assay

To generate infectious HIV-1 particles (LAI isolate), we used the supernatants of chronically infected ACH-2 cells as per our published method^61^. Briefly, ACH-2 cells were cultured overnight and were activated with PMA and TNF-α. The virus containing supernatant was collected by centrifugation and filtering through a 0.45 μm pore-size syringe filter^61^. The concentration of the virus was measured by the p24-specific enzyme-linked immunosorbent assay (ELISA)^91^. Transmigration of HIV-1 virions was tested in the HBMEC barrier model by adding HIV-1 particles to the upper chamber. Culture media from the bottom chamber were collected at different time points and tested for the presence of virus by measuring infectivity. Virus infectivity was determined by TZM-bl cell based luciferase reported assay^92^.

Transmigration of monocytic cells through the barrier of the HBMECs was tested by adding THP-1 monocytic cells to the upper chamber. As a function of time, transmigration of these cells to the lower chamber was measured by counting the number of cells in the lower chamber by trypan blue method. To measure effects of cocaine on transmigration, the barrier was treated with cocaine for 24 h. Then the barrier was washed prior to addition of THP-1 cells to the upper chamber. To test the effects of MMP, the HBMEC barrier was treated with the MMP inhibitor (GM6011) with or without cocaine followed by addition of THP-1 cells. Similarly, to probe effects of prolidase, the barrier was exposed to the prolidase inhibitor (CBZ-Pro) in the absence or presence of cocaine (50 μM). Thereafter, the barrier was washed with PBS and THP-1 cells were added to the upper chamber and transmigration was measured in the lower chamber.

### Statistical analysis

Data were expressed as mean ± SEM obtained from three independent experiments. Significance of differences between control and treated samples was determined by Student’s t-test or two-way ANNOVA wherever necessary. Values of p < 0.05 were considered statistically significant. Western blot band intensity for each time course or dose response treatment was normalized to loading controls.
